# Pylephlebitis Caused by Actinomyces Bacteremia

**DOI:** 10.7759/cureus.2887

**Published:** 2018-06-26

**Authors:** Omar Abughanimeh, Mohammad Tahboub, Yousaf Zafar, Mouhanna Abu Ghanimeh

**Affiliations:** 1 Internal Medicine, Kansas City School of Medicine, Kansas City, USA; 2 Internal Medicine, University of Missouri at Kansas City, Kansas City, USA; 3 Internal Medicine, University of Missouri Kansas City, Kansas City, USA; 4 Internal Medicine/Gastroenterology, Henry Ford Health System, Detroit, USA

**Keywords:** pylephlebitis, pyelophlebitis, portal vein thrombosis, actinomyces

## Abstract

Pylephlebitis is a thrombophlebitis of the portal vein or its branches, which usually occurs as a complication of intra-abdominal infections that are drained by the portal vein, most commonly as a result of diverticulitis or appendicitis. Diagnosis of pylephlebitis is achieved by visualizing a portal vein thrombosis in a patient with bacteremia or a recent intra-abdominal infection. Many microorganisms have been reported to cause this infection. However, Actinomyces has never been reported before as a cause of pylephlebitis. Here, we describe a case of a 56-year-old female who had pylephlebitis that was caused by Actinomyces bacteremia and was treated with intravenous antibiotics.

## Introduction

Pylephlebitis is a rare condition, which is also known as thrombophlebitis of the portal venous system [1]. It is considered as a complication of intra-abdominal infection of areas that are drained by the portal venous system, most commonly caused by diverticulitis or appendicitis [1-2]. The infection is usually polymicrobial, with *Escherichia coli*, Bacteroides, and Streptococci being the most commonly isolated organisms [3]. The presentation of pylephlebitis is nonspecific and usually consists of fever, abdominal pain, and fatigue [4-5]. Diagnosis is usually done by either Doppler ultrasonography or computed tomography (CT) that demonstrates a portal vein thrombus in the setting of bacteremia or a recent intra-abdominal infection [2, 5]. Antibiotics are the cornerstone of treatment for pylephlebitis [2]. However, the use of anticoagulation is not well defined in the treatment of this disease [1]. Treatment is crucial to prevent complications or death. Despite all the advances in diagnostic techniques and treatment, pylephlebitis still has a high mortality rate of 11%-32% [1-2, 5]. 

## Case presentation

A 56-year-old female, with a history of a repaired Tetralogy of Fallot and pulmonary embolism while on warfarin, presented with epigastric pain and melena. The patient was febrile (101.2℉) but hemodynamically stable and did not appear to be septic. Labs on admission are shown in Table 1.

**Table 1:** Vital signs and labs on presentation. T: temperature; HR: heart rate; BP: Blood pressure; O_2_ sat.: oxygen saturation; CBC: complete blood count; Na: sodium; K: potassium; Cl: chloride; AST: aspartate aminotransferase; ALT: alanine aminotransferase; ALP: alkaline phosphatase.

Vital signs: T: 101.2, HR: 102, BP: 148/84, O_2_ sat.: 99%	
CBC	
Hemoglobin	11.7 g/dL
White blood cells	16.1 x 10^3 ^cmm
Platelets	349
Chemistry	
Na	139 mmol/L
K	4.7 mmol/L
Cl	99 mmol/L
Creatinine	0.57 mg/dL
AST	26 U/L
ALT	30 U/L
ALP	113 IU/L
Total bilirubin	1.4 mg/dL
Lipase	14 U/L

**Figure 1:**
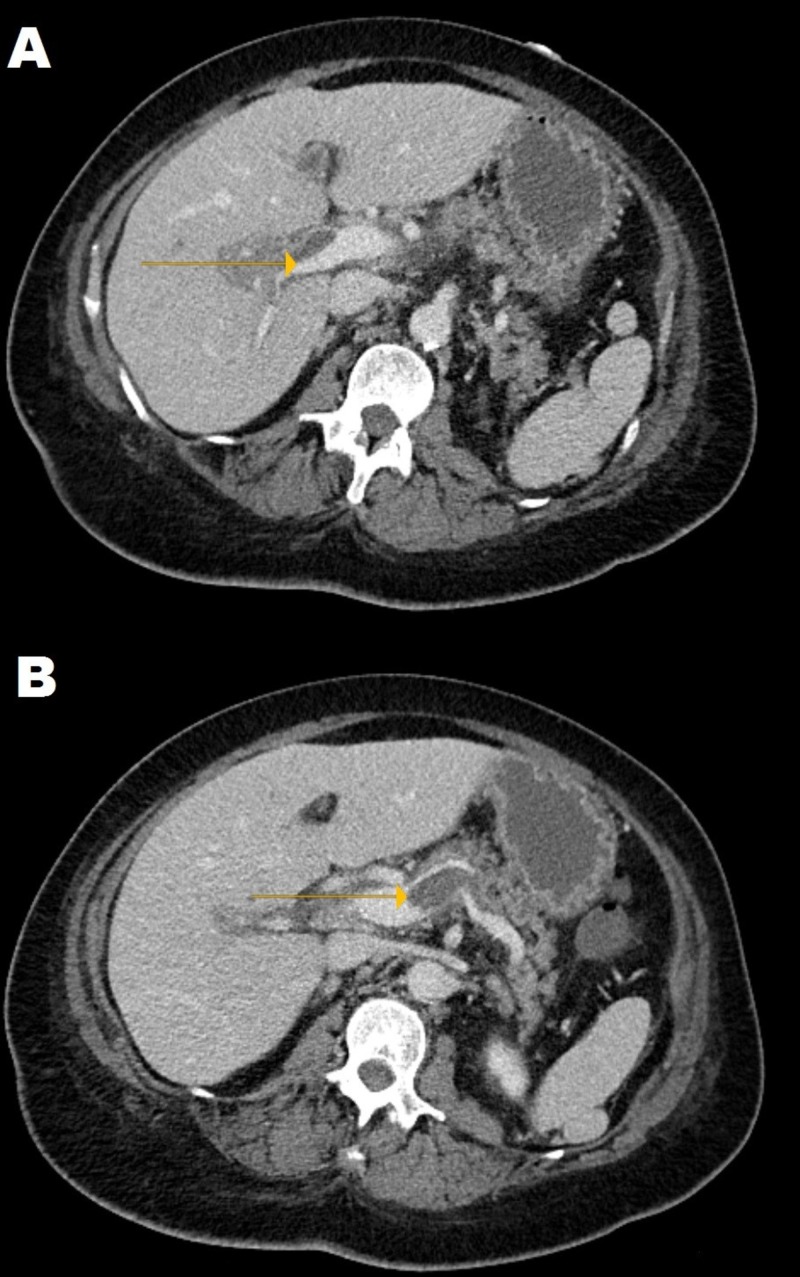
An abdominal computed tomography scan with contrast showing an acute portal vein thrombosis (A) extending into the splenic vein (B) and segmental branches of the right and left hepatic lobes.

The patient was given 10 mg of vitamin K intravenously and six units of fresh frozen plasma. The esophagogastroduodenoscopy (EGD) showed two nonbleeding duodenal arteriovenous malformations (AVMs). Her total bilirubin level increased to 3.0 mg/dL on day three of her hospital stay. An abdominal ultrasound (US) scan and a CT scan with/without contrast (Figure 1) showed acute portal vein thrombosis extending into the splenic vein and segmental branches of the right and left hepatic lobes. No abscesses or other sources of infection were noted.

**Figure 2:**
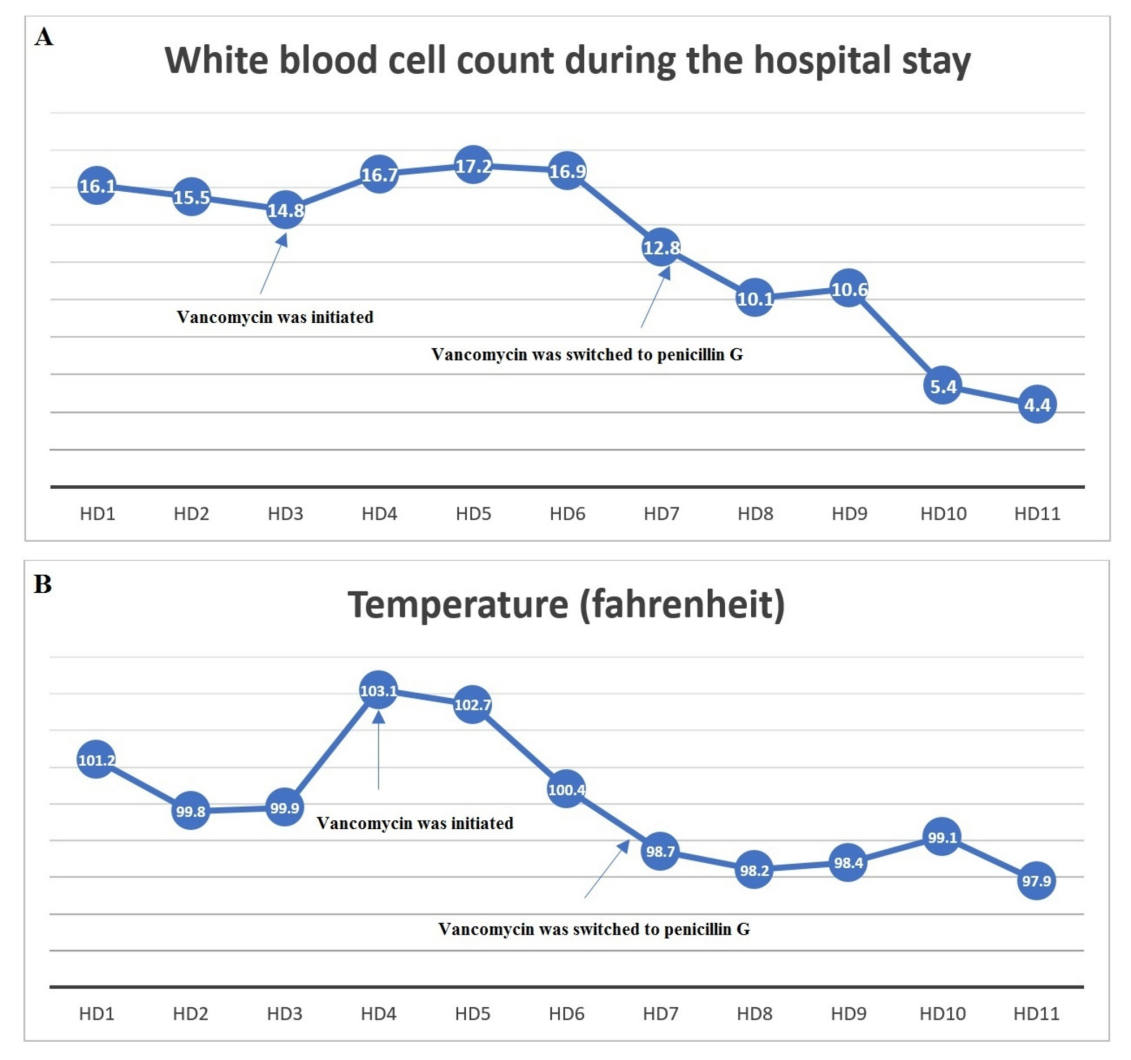
The patient’s white blood cell count (A) and temperature (B) trends during the hospital course.

The patient was started on 1 mg/kg enoxaparin daily (INR 1.5 on day three). The patient’s initial fever and leukocytosis were attributed to portal vein thrombosis; thus, no antibiotics were given, pending blood cultures. The next day, the blood cultures grew Gram-positive cocci and rods (*Micromonas miros* and *Actinomyces turicensis*, respectively). She was started on IV vancomycin. However, she continued to spike fevers with worsening leukocytosis (Figure 2). An echocardiogram did not show any valve vegetation. A tagged WBC scan showed no evidence of infection, making infective endocarditis unlikely. Her dental evaluation showed poor oral hygiene, multiple retained roots, pulpal necrosis, and mobile teeth. Repeated blood cultures grew *Actinomyces meyeri*. Both the hepatology and infectious diseases teams agreed this was likely a septic pylephlebitis secondary to Actinomyces bacteremia (likely stemming from the oral cavity). She was switched to IV penicillin G, after which her WBC count improved (Figure 2) and repeated blood cultures came back negative. She was discharged on IV ertapenem for six weeks followed by six weeks of oral amoxicillin and a follow-up appointment for oral surgery.

## Discussion

Pylephlebitis is a rare condition caused by an infected thrombosis of the portal venous system, and hence it is called suppurative thrombophlebitis [1]. It is considered as a complication of an intra-abdominal infection that is drained by the portal venous system, mostly as a result of diverticulitis or appendicitis [1-2]. It was first described in 1846 by Waller during an autopsy on a patient that had a case of hepatic abscess [1]. Pylethrombosis is different from pylephlebitis in that it is a thrombosis in the portal venous system without infection [2]. The incidence of pylephlebitis is unknown but thought to be close to 2.7 per 100,000 persons per year [1].

Pylephlebitis is commonly caused by infections in the regions that are drained by the portal system. The infection starts in small mesenteric veins, then extends to larger veins of the portal system and the liver [1, 3]. Another way for pylephlebitis to develop is through bacterial seeding of a pre-existing portal vein thrombosis [6]. Diverticulitis and appendicitis are the most common causes, with other causes including pancreatitis, inflammatory bowel disease, or other abdominal infections [1]. Pylephlebitis has also been reported to occur after some procedures such as CT, guided liver biopsy, hemorrhoidal banding, and gastric banding [5-7]. Hypercoagulable states or clotting factor deficiencies are considered as a risk factor for pylephlebitis; other risk factors are cirrhosis, recent abdominal surgery, malignancy, smoking, steroid use, and immobility [1].

The infection in pylephlebitis is usually polymicrobial; Bacteroides, *E. coli*, and Streptococci are the most common pathogens [3]. Plemmons et al. [2] reviewed 19 cases from 1979 to 1993 and found that diverticulitis was the most common cause, being responsible for 13 cases (68%). The same study showed that 88% of the patients had bacteremia and that Bacteroides, Gram-negative bacilli, and Streptococci were the most commonly isolated organisms. Kanellopoulou et al. [3] performed a retrospective literature review of all reported pylephlebitis cases in the English language literature from 1971 to 2009, and found 100 reported cases. Upon analysis of the etiology, diverticulitis was found to be the most common cause (30%), followed by appendicitis (19%), inflammatory bowel disease (6%), and pancreatitis (5%). Bacteremia was evident in 60% of the cases; a single microorganism was isolated in 47% of the cases, while two or more bacterial organisms were isolated in the rest. The most common pathogens were Bacteroides, *E. coli*, and Streptococci; none of these cases had Actinomyces. Choudhry et al. [1] performed a more recent retrospective study that included a chart review from 2002 to 2012, during which time they found 95 cases of pylephlebitis. While analyzing the causes, they found that pancreatitis was the most common cause, accounting for 31% of the cases, followed by diverticulitis (19%), peritonitis (15%), and intra-abdominal abscesses (13%). It is worth mentioning that this study was conducted in a tertiary center that had many referrals for pancreatic and hepatobiliary conditions, which may not be reflective of the general population. The same study showed that bacteremia was present in 34 patients (44%). The most common organism cultured was Streptococcus viridans, followed by *E. coli* and Bacteroides fragilis. Again, Actinomyces was not isolated in any of the cases.

Pylephlebitis is difficult to diagnose given its nonspecific presentation; usually patients will have fever, abdominal pain, nausea, jaundice, and hepatomegaly [1, 4-5]. Plemmons et al. [2] found that fever was the most common presenting symptom and was present in all their patients (19 cases), followed by abdominal pain which was found in 74% of the patients. Kanellopoulou et al. [3] found that the most common presenting symptom was fatigue (95%), followed by fever (86%) and abdominal pain (82%). The same study analyzed laboratory tests and found that leukocytosis was the most common finding (80%), followed by elevation of liver enzymes (69%), hyperbilirubinemia (55%), and anemia (55%).  

The diagnosis of pylephlebitis is often made using Doppler ultrasonography or CT that shows a portal vein thrombosis in a patient with bacteremia or intra-abdominal infection [2, 5].

Treatment of pylephlebitis is important to prevent complications; antibiotics form the cornerstone of treatment. The choice of antibiotic should be based on the culture results (either from blood or a surgical sample). It is recommended to prescribe at least four weeks of antibiotic therapy in a patient who does not have a visualized abscess, and six weeks for a patient who has liver abscess with considerations of drainage [2]. Another accepted regimen is to treat with two weeks of parenteral antibiotics followed by three to four weeks of oral treatment [8].

The use of anticoagulation in pylephlebitis is a controversial topic [1]. The purpose of anticoagulation is thought to be the prevention of the progression of thrombosis and the treatment of complications of portal vein thrombosis [3]. Kanellopoulou et al. [3] noted that early use of anticoagulation was associated with a decreased mortality rate and better recanalization. However, Plemmons et al. [2] did not find a statistically significant effect on mortality. Baril et al. [9] performed a retrospective study on 44 patients with pylephlebitis to assess the use of anticoagulation; they came to a conclusion that anticoagulation should be considered in patients with a hypercoagulable state due to a deficiency of clotting factors, cancer, or in the case of mesenteric vein involvement as the risk of infarction will be higher.

The use of invasive methods such as thrombectomy, catheter-directed thrombolysis, and systemic thrombolysis has been reported [1, 3]. For example, Sherigar et al. [4] described a case of a 59-year-old male who received parenteral antibiotics for nine days without improvement in symptoms or via imaging to CT scan; he was subsequently treated with a 10-mg bolus of alteplase followed by a tapered infusion over 24 hours, which resulted in resolution of symptoms thrombus itself, based on imaging, within 24 hours. In refractory cases, surgical thrombectomy or placement of a percutaneous drain into the portal vein might be necessary [1, 10]. However, it is worth mentioning that surgical thrombectomy has been reported to have higher recurrence rates [1].

As mentioned earlier, treatment is crucial to prevent complications or death. Pylethrombosis can lead to mesenteric ischemia, infarction, and can extend to other veins including superior mesenteric vein, splenic vein, or other nearby veins [1, 3]. Other known complications include the formation of hepatic abscesses, sepsis, and the development of portal hypertension [4,9]. Even with the use of antibiotics, pylephlebitis continues to have high mortality rates 11%-32% [1-2, 5].

Our case was presented in the American College of Gastroenterology annual meeting 2018 as an abstract [Abstract: Abughanimeh O, Tahboub M, Asif T, et al. I Have Not Heard About Septic Portal Vein Thrombophlebitis, Have You?. Am J Gastroenterol 2017; 112 (S1): S1278–S1279; DOI:10.1038/ajg.2017.321. PMID: 28981060. https://eventscribe.com/2017/wcogacg2017/ajaxcalls/PosterInfo.asp?efp=S1lVTUxLQVozODMy&PosterID=116013&rnd=0.4473327].

## Conclusions

Pylephlebitis is a rare condition, but it has been reported. It needs high clinical suspicion given its nonspecific presentation. It should be considered in cases of portal mesenteric venous thrombosis and fever. Early identification and initiation of antibiotics is crucial in decreasing mortality rate significantly. The role of anticoagulation in pylephlebitis is a controversial topic; further studies are needed to assess its benefits and risks in this disease.
